# Fabrication of Highly Efficient Perovskite Nanocrystal Light-Emitting Diodes via Inkjet Printing

**DOI:** 10.3390/mi13070983

**Published:** 2022-06-22

**Authors:** Taikang Ye, Siqi Jia, Zhaojin Wang, Rui Cai, Hongcheng Yang, Fangqing Zhao, Yangzhi Tan, Xiaowei Sun, Dan Wu, Kai Wang

**Affiliations:** 1 College of New Materials and New Energies, Shenzhen Technology University, Shenzhen 518118, China; 2 Guangdong University Key Laboratory for Advanced Quantum Dot Displays and Lighting, Shenzhen Key Laboratory for Advanced Quantum Dot Displays and Lighting, and Department of Electrical and Electronic Engineering, Southern University of Science and Technology, Shenzhen 518055, China; 12055011@mail.sustech.edu.cn (T.Y.); jiasiqi12@163.com (S.J.); 11849608@mail.sustech.edu.cn (Z.W.); cair@mail.sustech.edu.cn (R.C.); yanghc@mail.sustech.edu.cn (H.Y.); 11968010@mail.sustech.edu.cn (F.Z.); 11711611@mail.sustech.edu.cn (Y.T.); sunxw@sustech.edu.cn (X.S.); wangk@sustech.edu.cn (K.W.); 3 Department of Mathematics and Theories, Peng Cheng Laboratory, Shenzhen 518038, China

**Keywords:** inkjet printing, perovskite, nanocrystal, light-emitting diodes

## Abstract

As an effective manufacturing technology, inkjet printing is very suitable for the fabrication of perovskite light-emitting diodes in next-generation displays. However, the unsatisfied efficiency of perovskite light-emitting diode created with the use of inkjet printing impedes its development for future application. Here, we report highly efficient PeLEDs using inkjet printing, with an external quantum efficiency of 7.9%, a current efficiency of 32.0 cd/A, and the highest luminance of 2465 cd/m^2^; these values are among the highest values for the current efficiency of inkjet-printed PeLED in the literature. The outstanding performance of our device is due to the coffee-ring-free and uniform perovskite nanocrystal layer on the PVK layer, resulting from vacuum post-treatment and using a suitable ink. Moreover, the surface roughness and thickness of the perovskite layer are effectively controlled by adjusting the spacing of printing dots. This study makes an insightful exploration of the use of inkjet printing in PeLED fabrication, which is one of the most promising ways for future industrial production of PeLEDs.

## 1. Introduction

Metal halide perovskite is an outstanding light-emitting material due to its excellent optoelectronic properties including tunable bandgap, good color purity, and high photoluminescence quantum yield (PLQY) [1,2,3,4,5,6,7,8]. Based on these properties, the perovskite light-emitting diodes (PeLEDs) have achieved remarkable progress [9,10,11,12] in electroluminescence (EL) devices, which are highly efficient and highly luminescent. A typical PeLED is a multilayer device comprising electrodes, charge-transporting layers, and the emission layer. Perovskite nanocrystal is a promising candidate for the PeLED emission layer, as it has a higher PLQY, homogeneous fluorescence, and better film uniformity, compared with polycrystalline film perovskite [13,14]. Most of the emission layers in perovskite nanocrystal LEDs are deposited via the conventional spin-coating method, as perovskite nanocrystals are solution-processable and have good morphology control, but the resulting large amount of material waste (up to 98%) and a small fabrication area of spin-coating technique limits its application in future mass-scale industrial production [15].

Among various fabrication techniques, the inkjet printing technique demonstrates many advantages, including efficient use of material, being non-contact, high accuracy, and large area patterning capability [16]. Previous research demonstrates compatibility of inkjet printing for a wide variety of materials, ranging from quantum dots (QDs) [17,18], carbon nanotubes (CNTs) [19,20], metal nanoparticles [21,22], etc. [16,23]. As the perovskite nanoparticles can maintain stability under the colloidal state, the inkjet printing technique shows great potential to realize the scale-up application of perovskite material [24]. Currently, many researchers reported the fabrication of perovskite materials via inkjet printing on composite substrates to fabricate electronic devices [25,26,27]. However, most studies focused on the study of photoluminescence, and only a handful of researchers reported PeLEDs with high-efficiency EL with the use of inkjet printing [28,29,30].

Two key issues should be considered to fabricate a nanocrystal PeLED via inkjet printing. Firstly, printable ink should both disperse the perovskite nanocrystals and possess suitable viscosity and surface tension. Using the original solvent during synthesis for perovskite nanocrystals can avoid possible damage, but commonly used perovskite nanocrystal solvents (e.g., n-octane and n-hexane) are not suitable for inkjet printing due to their low viscosity and high evaporation rate, as a result of which stable flying inkjet droplets cannot be formed. Another critical issue is a highly uniform film without pinholes, coffee rings, and interfacial intermixing, all of the factors that help achieve good performance in PeLED devices [23,31]. This is a complex parameter optimization problem influenced by the fluid properties, spreading, and drying process of perovskite ink on the substrate. Recently, several studies reported inkjet-printed PeLED based on the use of nanocrystals; however, the reported external quantum efficiency (EQE) [28] and current efficiency [29] of the devices are relatively low, and there is still a large gap to improve the efficiency of the device.

In this study, we fabricated highly efficient PeLED by improving the quantity of the printed perovskite nanocrystal layer. Perovskite nanocrystals with a composition of FAPb_0_._7_Sn_0_._3_Br_3_ were chosen as the emitting material. A printable type of ink was developed based on binary solvents, which is suitable for perovskite nanocrystals dispersion and causes little damage to the underlying functional layer in PeLED. By adjusting print spacing and post-treatment, a very flat and smooth perovskite film with the root-mean-square (RMS) roughness of 0.7 nm was obtained. Consequent to the good quality of the fabricated inkjet-printed perovskite film, the PeLED device demonstrated outstanding performance, with an peak EQE of 7.9%, a maximum current efficiency of 32.0 cd/A, and a peak luminance of 2465 cd/m^2^. By exploring this technique, this study greatly contributes to one of the possible future industrial production methods of PeLEDs.

## 2. Experiments

### 2.1. Preparation

#### 2.1.1. Perovskite Nanocrystal Synthesis

The FAPb_x_Sn_1−x_Br_3_ perovskite nanocrystals were synthesized in the air at room temperature according to the study of Cai et al. [32]. By adjusting the composition ratio of Sn, the PLQY of perovskite nanocrystal was achieved as 97.5% when x was 0.2. The highest device performance was achieved when x was 0.3, with more matched energy levels and a high PLQY of 95.5%. The detailed synthesized process is shown below. First, 0.1 mmol FABr and 0.1 mmol of PbBr_2_ and SnBr_2_ mixture with different stoichiometric ratios (PbBr_2_: SnBr_2_ = 1 − x: x, x from 0 to 1 with step of 0.1) were dissolved in a 1 mL N, N-dimethylformamide (DMF) solution. Then, 200 μL oleic acid as well as 40 μL oleyl amine were added into this mixture successively to form a precursor. After intensively mixing the precursor, 400 μL precursor was injected into 10 mL chloroform. A yellow-greenish colloidal solution appeared after a slight blender of chloroform. Then, a 5 mL toluene/acetonitrile mixture (volume ratio 1:1) was added to the colloidal solution for further purification. After centrifuging at 7500 rpm for 4 min, the sediment was redisposed into 1 mL n-octane. The suitable size perovskite nanocrystals were separated via centrifugation at 4000 rpm for 2 min; the sediment contained large-sized perovskite nanocrystals, and the liquid supernatant was reserved as the targeted product.

#### 2.1.2. PeLED Device Fabrication

The substrates of PeLED devices were clean-patterned ITO glass with a sheet resistance of 20 Ω/sq. ITO glass was cleaned in soaped water, deionized water, and isopropanol, in turn, for 10 min using an ultrasonic cleaner, and then the cleaned and dry ITO glass was treated with O_2_ plasma for 300 s. After that, PEDOT: PSS (purchased from Lumtec(New Taipei City, Taiwan, China), filtered through a 0.22 μm filter) was spin-coated on the ITO glass at 4000 rpm for 45 s and baked at 130 °C for 15 min in the air. Other function layers were prepared in a N_2_ glove box (H_2_O < 0.1 ppm, O_2_ < 0.1 ppm). Briefly, a 1.5 nm MoO_3_ layer was deposited on the (poly(3,4-ethylenedioxythiophene): poly (styrene sulfonate) (PEDOT: PSS) layer via thermal evaporation in a high vacuum chamber (<5 × 10^−4^ Pa) with a speed of 0.004 nm·s^−1^. After spin-coating a poly(9-vinylcarbazole) (PVK, bought from Lumtec, 100,000 molecular weight) solution (8 mg/mL in chlorobenzene) at 3000 rpm for 45 s and baking at 120 °C for 10 min, perovskite layers were deposited via inkjet printing with different drop steps. A Microfab JETLAB 2 printer equipped with a piezoelectric-driven inkjet nozzle (diameter: 40 μm) was used to print the perovskite film in the glove box, and the volume of a single droplet was about 60 pL. The perovskite film was treated in vacuum to accelerate the drying of ink. Finally, the device was transferred into a vacuum chamber (<5 × 10^−4^ Pa) for later functional layers. TPBi (45 nm), LiF (1 nm), and cathode Al (100 nm) were deposited via thermal evaporation, with speed rates of 0.05 nm·s^−1^, 0.005 nm·s^−1^, and 0.3 nm·s^−1^, respectively. Then, the device was taken out and tested in the air at room temperature without any packaging steps.

### 2.2. Characterization

The contact angle was tested using an instrument (JC200C1, Poareach, Shanghai, China) at room temperature. The absorption spectrum in the ultraviolet–visible band was measured using a PerkinElmer LAMBDA 950 spectrophotometer. The surface morphology of perovskite film was characterized via atomic force microscopy (AFM, Bruker Multimode 8) and field-emission scanning electron microscopy (FE-SEM, ZEISS G300). The PL spectrum and PLQY of perovskite nanocrystals were measured using an absolute PLQY spectrometer (Hamamatsu Quantaurus-QY C11347-11) with an excitation of 365 nm from a xenon lamp. The PL image of the perovskite dot array was taken with an optical microscope (Olympus CX41) under ultraviolet light of 365 nm. The 365 nm UV light can satisfy the excitation requirement as found in a typical excitation light for green perovskite nanocrystals [2,33].

The EL spectrum was measured using a fiber optic spectrometer (Ocean Optics USB 2000) in front of the luminance area. The stability of the EL spectrum was verified through the measurement of PeLED devices under different levels of bias. The current density–luminance–voltage curves of PeLEDs were measured in the air at room temperature with a PIN-25D silicon photodiode, and a dual-channel Keithley 2614B source meter. In this test system, a 4 mm^2^ emitting area was considered a Lambeau luminaire. By combining the luminous power received by photodiodes and the EL spectrum, the luminance can be calculated with the human visual photonic curve. In this study, 16 PeLED devices were used to calculate the standard deviation (0.991) for the EQE test.

## 3. Results and Discussions

**Figure 1:**
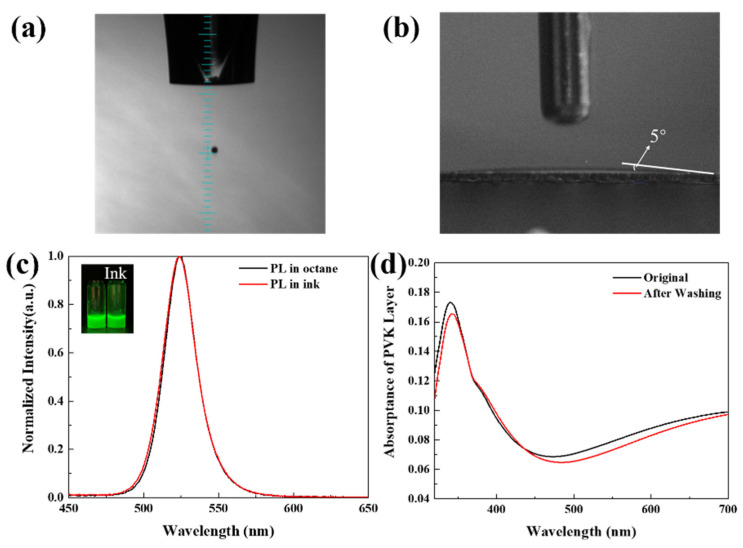
(**a**) Stable perovskite nanocrystal ink droplet existing from the nuzzle; volume of the droplet was around 60 pL; (**b**) contact angle of a perovskite nanocrystal ink drop on the PVK layer; (**c**) the PL spectra of the perovskite nanocrystal in octane and ink, with the inset showing the yellow-greenish images of the two samples under the excitation of 365 nm ultraviolet light; (**d**) ultraviolet–visible absorptance spectrum of initial and ink without perovskite nanocrystal washed PVK layer.

To obtain printable perovskite ink, the prepared perovskite nanocrystals were dispersed in a binary ink system (dodecane and octane) [31]. The viscosity and surface tension of these solvents are shown in Table 1. The main solvent of dodecane was used to achieve suitable viscosity for stable ink droplet formation and good dispersion for perovskite nanocrystals. As Figure 1a shows, a stable droplet without satellite dropped from the nuzzle, demonstrating the printability of perovskite nanocrystal ink. As reported in a previous study [23], a highly uniform printed film is crucial for multilayer LED devices. In inkjet printing, a small contact angle of ink on the substrate is required, as a large contact angle increases the difficulty of morphology control [17]. As shown in Figure 1b, the drop had a small contact angle of about 5° on the PVK layer, which results from the hydrophilic property of the main solvent used in the ink for PVK fabrication [33]. This benefits the formation of a uniform perovskite emission layer [34]. The comparison of PL spectra shown in Figure 1c proves that, compared with the original solution, the ink did not significantly affect the produced perovskite nanocrystal. The comparisons of TEM images of the dispersion of perovskite nanocrystal in the original solvent and ink are shown in Supplementary Figure S1, which proves the good dispersion of perovskite nanocrystals in the ink. Additionally, layer interference needs to be considered, where the underlying layer can be damaged by the solvent of the upper layer. The problem is more severe in inkjet printing, as the resting time of the solution is much longer than that in the spin-coating process. In the spin-coating process, a large proportion of solution is thrown away and quickly evaporated under high-speed rotation in less than 1 min. In inkjet printing, the evaporation time for the solution is more than 10 min. Considering the above issue, the toluene in our previous study [31] was replaced by octane to avoid the interference of toluene on the hole-transporting layer (PVK), which would cause serious layer interference between PVK and the emitting layer. To determine whether the composite-solvent ink would damage the underlying PVK layer, the absorptance of PVK was recorded after three times washing with a solution with the same composition of ink as that shown in Figure 1d. The absorptance spectrum showed little difference after washing with pure ink without perovskite nanocrystals. In short, the ink can be used to print a perovskite nanocrystal film on the PVK layer, which is the prerequisite for PeLED fabrication via inkjet printing.

**Figure 2:**
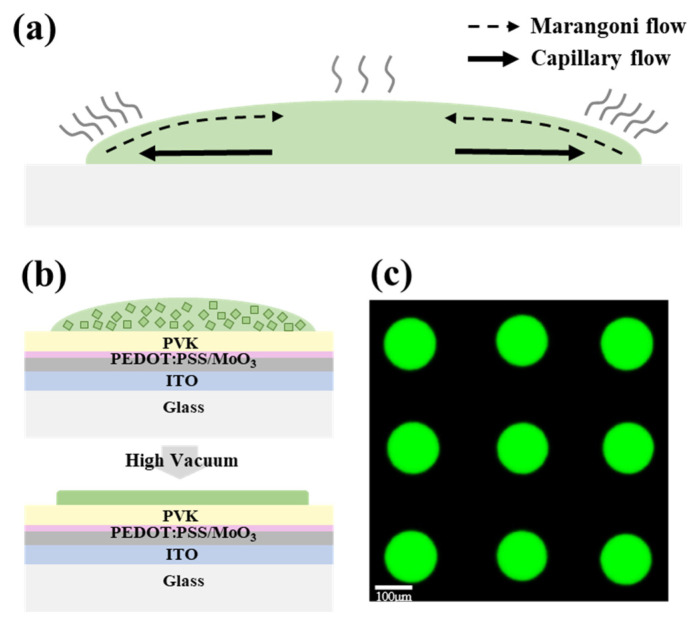
(**a**) The drying process of a single perovskite nanocrystal droplet on substrate; (**b**) schematic of a perovskite dot array on a multilayer (PVK/PEDOT:PSS/MoO3/ITO/Glass) substrate, the samples of which were then transferred to a high vacuum chamber to remove solvent; (**c**) image of the uniform, printed perovskite nanocrystal dots array under optical microscope with an excitation light of 365 nm. The scale bar is 100 μm.

To fabricate a perovskite nanocrystal film of high quality via inkjet printing, the coffee-ring effect during evaporation of the solvent should be solved. As shown in Figure 2a, in the drying process of a single droplet on the substrate, the evaporation speed of solvent at the edge of the droplet is different from the evaporation speed in the center [34], and the solute (perovskite nanocrystal) flows to the edge via capillary flow to form a rough surface, which is called the coffee-ring effect. To suppress the coffee-ring effect, the balance between the capillary flow and Marangoni flow has been investigated in our previous work [31]. A high-viscosity ink causes a weak capillary flow, which can suppress the migration of nanocrystal to the edge. In our binary solvent ink, the introduction of octane adjusted the viscosity to an optimal value, corresponding to a Marangoni flow that would simply offset the capillary flow to suppress the coffee-ring effect, due to the surface tension gradience on the surface of the droplet [31,35,36], which is because of the difference in surface tension of octane and dodecane, as shown in Table 1. Meanwhile, according to a simulation conducted by Park et al. [34], the small contact angle of the droplet on the PVK layer also suppressed the coffee-ring effect, because the droplet with a smaller contact angle spread more widely, and the solvent evaporated faster under high-vacuum post-treatment. As shown in Figure 2b, we printed a perovskite dot array on a multilayer substrate, which was fabricated via spin-coating PEDOT: PSS and PVK with a MoO_3_ interface layer via thermal evaporation on the patterned ITO glass, and the fabricated substrate can be directly used for PeLED devices. After inkjet printing, the samples were transferred into a high vacuum (<10^−4^ pa) chamber to remove the solvent. After vacuum annealing for 20 min, the coffee-ring-free and uniform dots array was formed, with a diameter of about 150 μm, as shown in Figure 2c. Every single dot connected with the adjoining dots when the spacing was reduced, to form a pinhole-free and uniform perovskite film.

**Figure 3:**
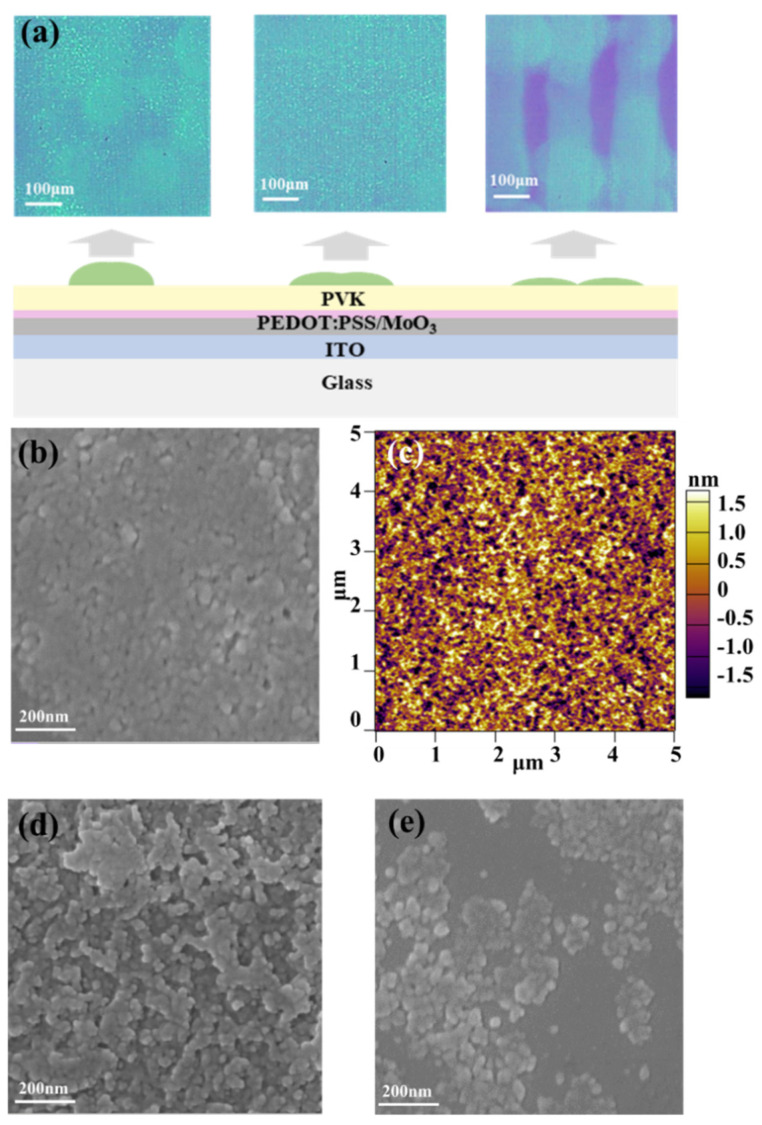
(**a**) Different film morphologies with different print spacing (from left to right, corresponding to 20, 60, and 100 μm spacing); (**b**) SEM image of the uniform, continuous perovskite film fabricated via inkjet printing with a spacing of 60 μm; (**c**) a 5 μm × 5 μm AFM image (RMS roughness: 0.7 nm) and SEM image (scale bar: 200 nm) of printed perovskite nanocrystal film with a printed spacing of (**d**) 20 μm and (**e**) 100 μm.

Although the optimal single dots were printed, dot spacing had to also be optimized because the perovskite film is formed by the connection of the dots via the inkjet printing process. The smooth, compact, and pinhole-free perovskite emissive films were well printed on the PVK layer with optimal thickness, which benefits the performance of PeLED. Here, we fabricated a thin perovskite film with different print spacings from 20 to 100 μm. The morphology characterization results of printed perovskite continuous films are shown in Table 2 and Figure 3. As shown in Table 2, a thicker perovskite emission layer was formed when the print spacing was decreased, and the optimal RMS roughness (0.7 nm) was obtained with a spacing of 60 μm. The comparative PL images of perovskite films with different print spacings under a fluorescence microscope are shown in Figure 3a. These results showed that a spacing of 60 μm was the best parameter to obtain a printed perovskite film with a pinhole-free and uniform perovskite film morphology. The AFM and SEM images of perovskite film with a 60 μm step are shown in Figure 3b,c, which indicate that a uniform and pinhole-free perovskite film was formed with a print spacing of 60 μm. When the print spacing was set overly large (100 μm), an ultrathin perovskite film with pinholes would result, leading to a serious current leakage, as shown in Supplementary Figure S2, which is harmful to PeLED. A small print spacing of 20 μm increased the thickness of the film, which can suppress the current leakage but cause poor morphology. The morphology of printed perovskite film with a small spacing (20 μm) and a large spacing (80 μm) using SEM are shown in Figure 3d,e. The gap can be observed in the SEM image with 80 μm print spacing, and the value of RMS of it was larger than that of 60 μm because the perovskite film could not cover the PVK layer well. When the print spacing decreased to 20 μm, the morphology quality seriously decreased due to the aggregation and overlapping of perovskite nanocrystal, leading to large roughness, as shown in Figure 3d. An ultralow root-mean-square (RMS) roughness of less than 1 nm is beneficial in enhancing the performance of the subsequent device.

**Figure 4:**
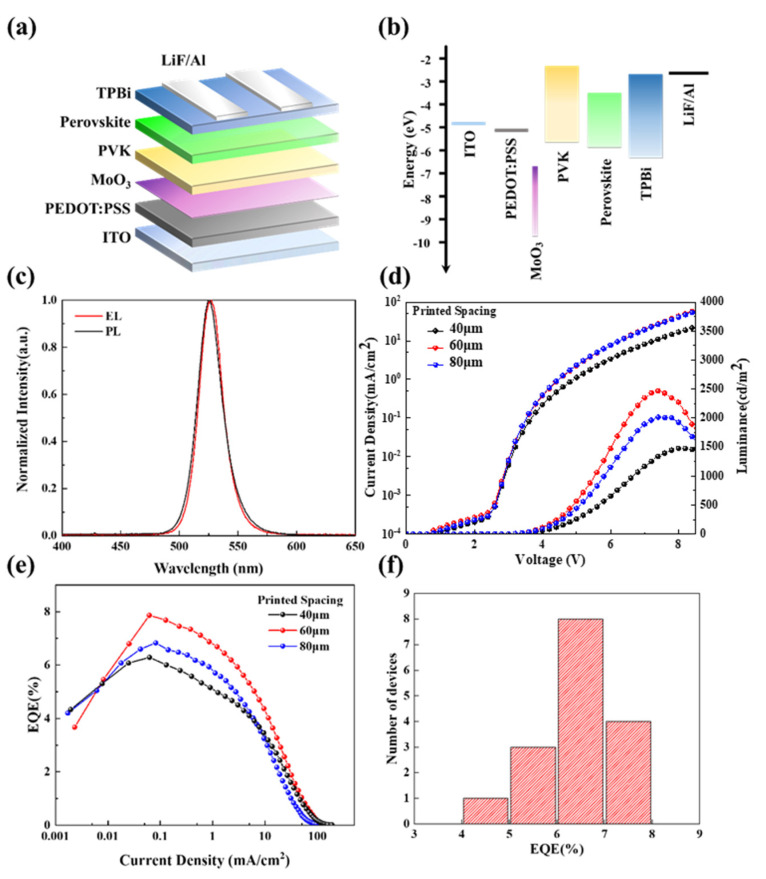
(**a**) Schematic of PeLED device structure; (**b**) energy band alignment of each layer in PeLED; (**c**) EL (at 5 V bias) and PL spectrum of perovskite nanocrystal fabricated via inkjet printing. (**d**) current density–luminance–voltage curve; (**e**) EQE–current density curve with different printed steps (40, 60, 80 μm); (**f**) histogram of maximum EQE for 16 inkjet-printed PeLED devices.

Printed PeLEDs were fabricated based on the perovskite films, with print spacing of 20, 40, 60, and 80 μm. The device schematic and band structure are shown in Figure 4a,b. The glass substrates with transparent ITO electrode patterns were used for PeLED fabrication due to the bottom-emitting device structure. The perovskite layer was sandwiched between other function layers including the 2,2′,2″-(1,3,5-Benzinetriyl)-tris(1-phenyl-1-H-benzimidazole) (TPBi, as the electron-transporting layer) and the PVK (as the hole-transporting layer), and the alumina as cathode, with 1 nm LiF modification, and indium tin oxide (ITO) as the anode. An ultrathin MoO_3_ layer was added between the PEDOT: PSS (served as a hole-injection layer) and the PVK acted as an electrical diploe layer. Due to the deep conduction band level of MoO_3_, a positive electric field could be formed across the PEDOT: PSS/MoO_3_/PVK, which can enhance hole injection and achieve a better charge balance in this device structure [36]. Figure 4c shows the pure PL and EL spectra with full width at half-maximum (FWHM) of 23 nm, and there was no emission from other function layers and interfaces, which means the recombination of electron and hole was limited inside the perovskite layer. The performance levels of devices with different printed steps are shown in Figure 4d,e. The comparison of spin-coating and inkjet printed devices performance are shown in Supplementary Figure S3. The key issue for achieving good performance in the device here is the quantity and thickness control of the inkjet-printed perovskite film. The thickness directly corresponds to the number of overlapped printed perovskite layers, and the thickness increases with the decrease in the print spacing. There are defects in perovskite nanocrystals [37], as well as at the interfaces between the emission layer and charge-transporting layers [13], which trap the charge and decrease the performance of these devices. A thicker perovskite layer has a relatively high tolerance for the interface trap, as there are more recombination zones, compared with a thinner perovskite layer, but the diffusing lengths (determined by traps of the perovskite nanocrystal) of carriers limit the increase in the thickness of emission layer. Therefore, when the spacing decreased from 80 to 40 μm, as shown in Figure 4d, the device presented a decreased current density due to the increased resistance with a thicker perovskite layer. Moreover, the perovskite film also became rougher with an increased thickness by overlapping multiple perovskite layers, and the excessively high roughness of perovskite film disabled the function of the EL device when the spacing decreased to 20 μm, as summarized in Table 3. When the spacing increased, as shown in Figure 3a, the perovskite film became discontinuous, thus causing current leakage, as shown in Supplementary Figure S4, and deteriorating the emitting performance of the device; therefore, the optimal spacing was determined as 60 μm, with the highest EQE of 7.9% and maximum luminance of 2465 cd/m^2^.

## 4. Conclusions

In summary, we fabricated a highly efficient PeLED via inkjet printing, by improving the quality and controlling the thickness of the perovskite film. The damaging influence of the ink on the underlying PVK layer was eliminated by adjusting the components of the ink. Meanwhile, the small contact angle of high-viscosity ink with PVK and vacuum annealing suppressed the coffee-ring effect to improve the quality of the perovskite layer. Continuous perovskite films were formed when the print spacing was reduced, and each dot connected to the adjacent dots. By controlling the thickness using an inkjet printer, the device with a spacing of 60 μm showed a maximum EQE of 7.9%, a peak current efficiency of 32.0 cd/A, and a peak luminance of 2465 cd/m^2^. According to Table 4, this device is one of the best performance inkjet-printed PeLED devices. This study revealed the considerable application potential of printed perovskite nanocrystals, in both photoluminescence and EL fields. The application of an automatic inkjet printing system can save manpower and provides a simpler way for device optimization, with fewer parameters, which can be combined with high-throughput optimization in the future.

## Figures and Tables

**Table 1:** Detailed data of solvents and our mixed ink.

Type of Solution	Molecular Weight	Boiling Point (°C)	Vapor Pressure (mmHg at 25 °C)	Viscosity (mPa·s at 25 °C)	Surface Tension (mN/m)
**Octane**	**114.23**	**125**	**14.1**	**0.51**	**21.14**
**Dodecane**	**170.33**	**216**	**0.135**	**1.48**	**25.44**
**Mixed Ink**	**/**	**/**	**/**	**1.25**	**23.3**

**Table 2:** Morphology performance of inkjet-printed perovskite film using different steps.

Steps (μm)	RMS Roughness (nm)	Thickness (nm)
**20**	**4.9**	**34.92**
**40**	**1.8**	**26.07**
**60**	**0.7**	**23.84**
**80**	**0.9**	**18.59**

**Table 3:** Performance of PeLED with different printing steps.

Steps (μm)	Turn-On Voltage (V)	Max. Current Efficiency (cd/A)	Max. EQE (%)	Max. Luminance (cd/m^2^)
**20**	**/**	**/**	**/**	**/**
**40**	**3.0**	**24.7**	**6.2**	**1476**
**60**	**3.0**	**32.0**	**7.9**	**2465**
**80**	**3.0**	**26.8**	**6.8**	**2017**

**Table 4:** Summary of inkjet-printed PeLEDs device performance.

Year	Emission Layer	Max. Luminance (cd/m^2^)	Max. Current Efficiency (cd/A)	Max. EQE (%)	Ref.
2020	FA_0_._3_Cs_0_._7_PbBr_3_ quantum dots	1233	10.3	2.8	[28]
2020	MAPbBr_3_	4000	<0.9	/	[29]
2021	CsPbBr_3_ quantum dots	10,992	8.67	3.03	[38]
2021	PEA_2_Cs_n−1_PbnBr_3n+1_ quasi-2D	3640	31.5	9.0	[30]
**This work**	**FAPb_0_._7_Sn_0_._3_Br_3_ nanocrystals**	**2465**	**32.0**	**7.9**	**/**

## Data Availability

Not applicable.

## Supplementary Materials

The following are available online at https://www.mdpi.com/article/10.3390/mi13070983/s1, Figure S1: TEM image of perovskite nanocrystal, Figure S2: Current density of printed PeLED devices with different print spacing, Figure S3: Device performance comparison between devices fabricated via inkjet printing and spin-coating, Figure S4: Luminance against the operation time of inkjet-printed PeLED.
