# Supplementary material for: Fabrication of Highly Efficient Perovskite Nanocrystal Light-Emitting Diodes via Inkjet Printing
Source: Micromachines (Basel). 2022 Jun 22;13(7):983. doi: 10.3390/mi13070983 (PMC9319064; doi:10.3390/mi13070983)
Supplement: Supplementary file 1 [file micromachines-13-00983-s001.zip › micromachines-1757004-supplementary.pdf]

micromachines

Article

Supporting Materials of Highly Efficient Perovskite Nanocrystal Light-Emitting Diodes via Inkjet Printing

Taikang Ye [2,†], Siqi Jia [2,3,†], Zhaojin Wang [2], Rui Cai [2], Hongcheng Yang [2], Fangqing Zhao [2], Yangzhi Tan [2] , Xiaowei Sun [2], Dan Wu [1,*] and Kai Wang [2]

[1] College of New Materials and New Energies, Shenzhen Technology University, Shenzhen 518118, China
[2] Guangdong University Key Laboratory for Advanced Quantum Dot Displays and Lighting, Shenzhen Key Laboratory for Advanced Quantum Dot Displays and Lighting, and Department of Electrical and Electronic Engineering, Southern University of Science and Technology, Shenzhen 518055, China
[3] Department of Mathematics and Theories, Peng Cheng Laboratory, Shenzhen, 518038, China
* Correspondence: wudan@sztu.edu.cn
† These authors contributed equally to this work.

Citation: Ye, T.; Jia, S.; Wang, Z.; Cai, R.; Yang, H.; Zhao F.; Tan, Y.; Sun, X..; Wu, D.; Wang, K. Fabrication of Highly Efficient Perovskite Nanocrystal Light-Emitting Diodes via Inkjet Printing . *Micromachines* **2022**, *12*, 983. https://doi.org/10.3390/mi13070983

Academic Editor: Jinn-Kong Sheu

Received: 20 May 2022
Accepted: 16 June 2022
Published: 22 June 2022

Publisher's Note: MDPI stays neutral with regard to jurisdictional claims in published maps and institutional affiliations.

Copyright: © 2022 by the authors. Submitted for possible open access publication under the terms and conditions of the Creative Commons Attribution (CC BY) license (http://creativecommons.org/licenses/by/4.0/).

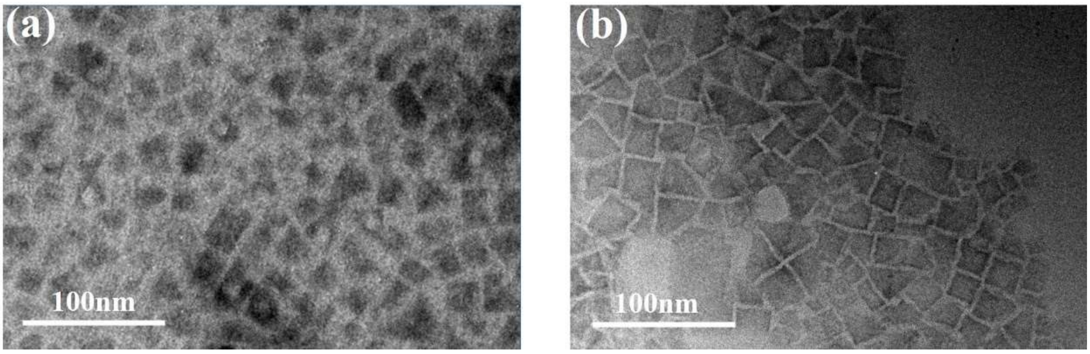

Figure S1. (**a**) TEM image of perovskite nanocrystal in ink; (**b**)TEM image of perovskite nanocrystal in initial solution.

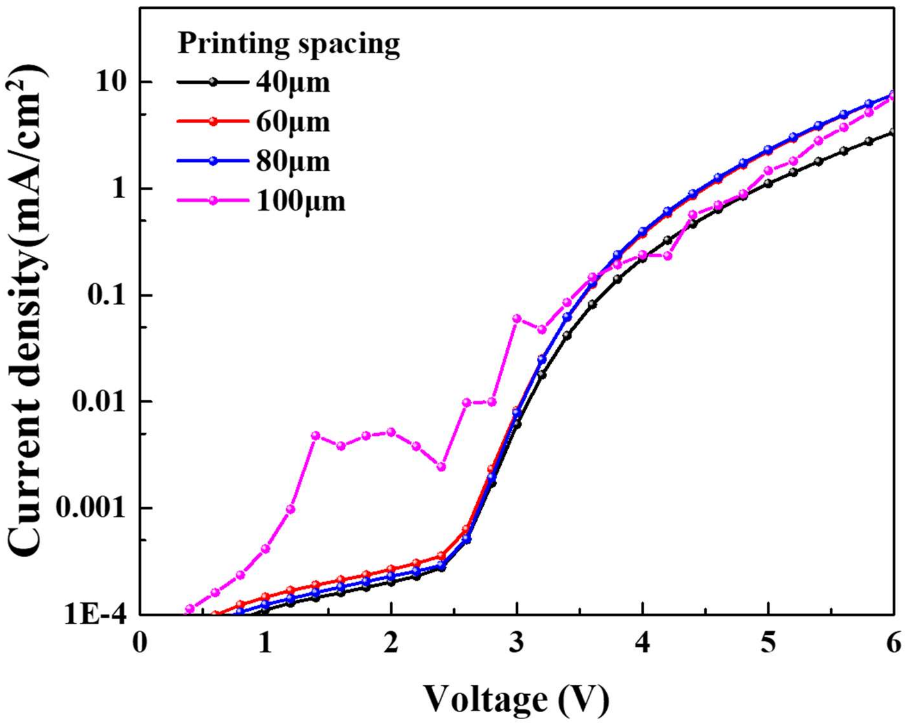

Figure S2. Current density of printed PeLED devices with different print spacings of 40, 60, 80, and 100 μm at low voltage. At a print spacing of 100μm, obvious current leakage was found between 1 and 3V.

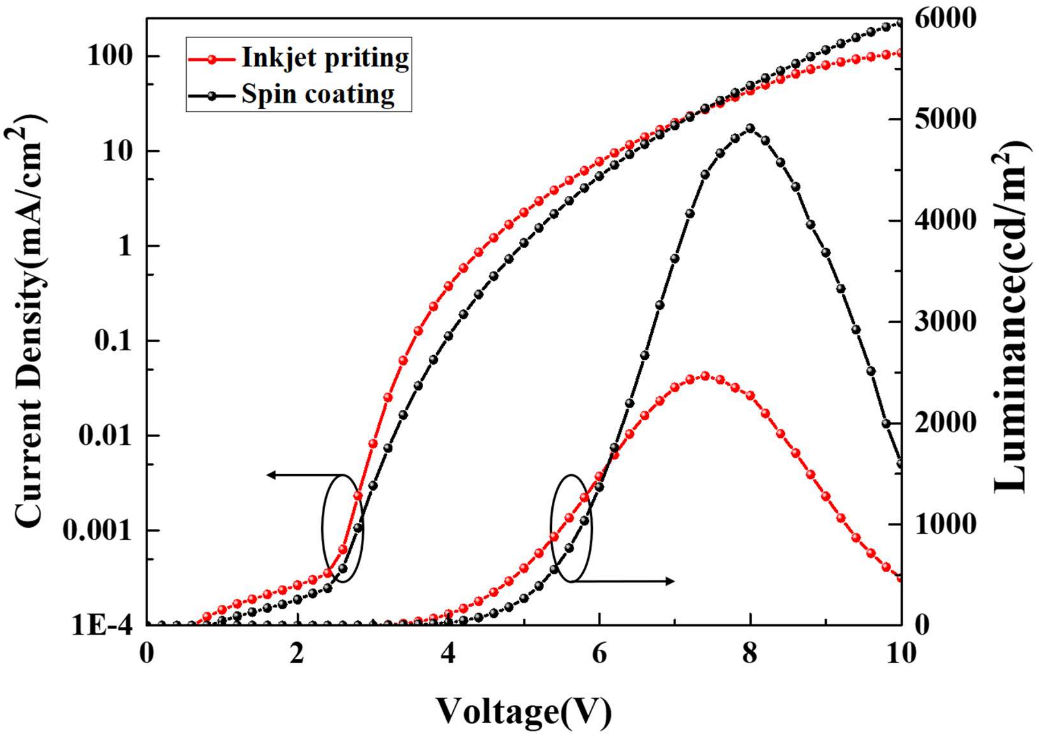

Figure S3. Device performance comparison between devices fabricated via inkjet printing and spin coating. As shown in this figure, the inkjet printing device had a lower turn-on voltage and higher current density when the applied voltage was lower than 6V, but the spin-coating device had a higher luminance than the inkjet-printed device. The possible reason for the performance difference is the slight dissolution of PVK layer due to the long residence time of ink. It may dissolve the PVK layer slightly and increase the contact area between the PVK and perovskite layer. This benefitted the carrier injection and reduced the turn-on voltage at a lower external voltage (<6V). However, the roughness of PVK layer increased at the same time, and more interface defects would therefore be generated, which would accelerate device degradation at high external voltage (>6V).

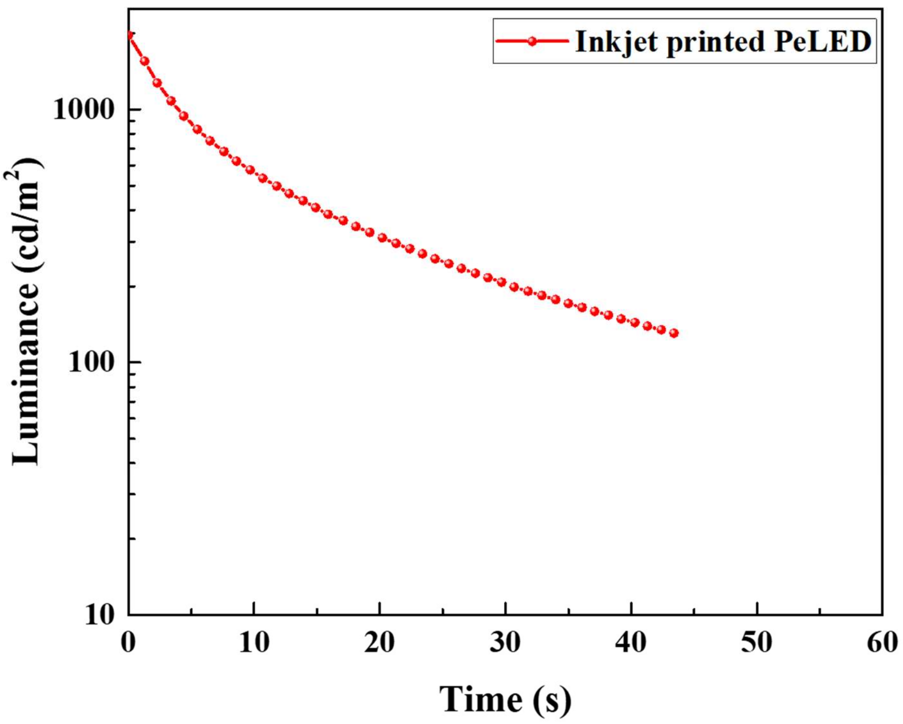

Figure S4. Luminance against the operation time of inkjet-printed PeLED under a constant current density of 10 mA/cm² and initial luminance of 2000 cd/m².
